# Association Analyses Between the *NPPB*:rs198389 Gene Polymorphism, NT-proBNP Serum Concentrations and Phenotypic Features in Patients with Heart Failure

**DOI:** 10.3390/genes17040454

**Published:** 2026-04-14

**Authors:** Anna Gorący-Rosik, Jakub Rosik, Klaudyna Lewandowska, Iwona Gorący, Andrzej Ciechanowicz

**Affiliations:** 1 Department of Clinical and Molecular Biochemistry, Pomeranian Medical University, 70-111 Szczecin, Poland; ania.goracy@gmail.com (A.G.-R.); klaudyna.lewandowska@pum.edu.pl (K.L.); iwona.goracy@pum.edu.pl (I.G.); 2 Department of Physiology, Pomeranian Medical University, 70-111 Szczecin, Poland; jakubrosikjr@gmail.com

**Keywords:** heart failure, B-type natriuretic peptide, gene polymorphism

## Abstract

Background: Heart failure (HF) is a complex disease and one of the major causes of morbidity and mortality in the world. Increased B-type natriuretic peptide (BNP) levels have been associated with HF. The *NPPB*:rs198389 (c.-381T > C) promoter polymorphism has been found to modulate BNP levels. Aim: To investigate possible associations among the *NPPB*:rs198389 polymorphism, N-terminal pro-BNP (NT-proBNP) concentrations, and phenotypic features in Polish patients with HF. Methods: The study group comprised 250 patients with HF. Genomic DNA was extracted from blood, and genotyping was performed using PCR-RFLP. Results: There were no significant differences in the distributions of *NPPB* genotypes or alleles between HF females and HF males. Except for body height, there were no significant differences in phenotypic features among HF patients regarding *NPPB*:rs198389 genotypes. There were also no significant differences in the distributions of either *NPPB*:rs198389 genotypes or alleles across NT-proBNP concentration terciles. However, age, left-ventricular-mass index, C-reactive-protein levels, serum-creatinine concentrations, and the incidence of myocardial infarction, left ventricular hypertrophy, or reduced ejection fraction (EF) were significantly lower in patients from the lower tercile (LT) than in patients from the middle and/or upper terciles. EF and the frequency of preserved EF in LT patients were significantly higher than those from other terciles. Conclusions: Our results did not confirm associations between *NPPB*:rs198389 and NT-proBNP serum concentrations or clinical phenotypes in Polish patients with HF.

## 1. Introduction

The burden of heart failure (HF) is continuing to acquire greater significance worldwide. Cardiovascular diseases (CVDs) remain the main causes of death in Poland, with HF as one of the prevailing CVDs, according to data from the Polish Central Statistical Office and the Ministry of Health. The number of HF patients in Poland has, in general, increased over the years and, despite enormous progress in HF management, this remains a challenge for the healthcare system in Poland, as well as in other countries [1]. With an aging population, the incidence of the illness is likely to increase further. Furthermore, the wide prevalence of other risk factors among all age groups, such as hypertension (HTN), obesity, elevated plasma lipid levels, or diabetes mellitus (DM), has contributed to an increase in the disease. Coronary artery disease (CAD) is a major cause of HF alongside heart valve disease, cardiomyopathies, or arrhythmias [2]. The development of HF is also influenced by neurohormonal and genetic factors, and, therefore, extensive research has pursued genetic testing, among other approaches, to better understand the mechanisms underlying HF development. Novel HF therapies continue to be created.

The diagnosis of heart failure is based on symptoms and/or signs secondary to structural and/or functional cardiac abnormality, together with elevated natriuretic peptide levels and/or signs of pulmonary or systemic congestion during examination or in subsequent tests. The presence of symptoms or signs of the disease is crucial for the diagnosis of chronic HF. According to the latest classification, HF may be categorized using the left ventricular ejection fraction (EF). This includes HF with reduced ejection fraction (HFrEF) with EF ≤ 40%; HF with mildly reduced ejection fraction (HFmrEF) with EF 41–49%; and HF with preserved ejection fraction (HFpEF) with EF ≥ 50% [3].

Natriuretic peptides play a crucial role in regulating the cardiovascular system. By exhibiting diuretic and natriuretic effects and mediating vasodilatation, they regulate blood pressure (BP) and body fluid volume. They exert anti-hypertrophic and anti-fibrotic effects, inhibit the activation of the renin–angiotensin–aldosterone system and sympathetic nervous system, and act by binding to the natriuretic peptide receptor (NPR). B-type natriuretic peptide (BNP) and N-terminal pro–B-type natriuretic peptide (NT-proBNP) are synthesized in the heart ventricles in response to volume and pressure overload. Their serum levels are low in healthy individuals, but elevated levels are reported in patients with HF. According to American and European guidelines, BNP and NT-proBNP are valuable biomarkers for diagnosing HF and assessing its severity. What is more, they may serve as prognostic tools in the course of the disease [4,5,6]. BNP and NT-proBNP may be useful in choosing a treatment strategy, as their levels have been shown to decrease during HF therapy. Hence, BNP-guided therapies could improve clinical outcomes if a decline in BNP levels occurs [7]. Additionally, malfunctions in the natriuretic peptide systems have been associated with atherosclerosis, thrombosis, and myocardial ischemia [8].

The *NPPB* gene, located on chromosome 1, encodes proBNP, which is then cleaved into BNP and the inactive fragment NT-proBNP. Some earlier studies have shown that polymorphism of the *NPPB* gene may be related to various diseases, such as DM, HTN, myocardial infarction (MI), and/or HF [9]. In particular, the rs198389 polymorphism has been recognized as a functional variant in the gene’s promoter region, disrupting a transcriptionally active enhancer box [10]. Seidelmann et al. showed that rs198389 was associated with higher circulating levels of NT-proBNP in the general population, including White and Black subjects, potentially due to higher activity in the promoter region of the *NPPB* gene. In fact, they also demonstrated a connection between the minor C allele of rs198389, lower BP, and decreased cardiovascular mortality [11]. Canone et al. investigated subjects at risk for HF from the STOP-HF study. They showed a significant association between the minor allele of rs198389 and higher BNP levels, a lower risk of HTN, and new left-ventricular systolic dysfunction. Furthermore, after five years of observation, carriers of the C allele demonstrated a decreased risk of major adverse cardiovascular event (MACE) and presented less favorable clinical phenotypes and outcomes in contrast to those who lacked this BNP genetic variant [12]. In contrast, in the FINRISK study, there was no association between the rs198389 polymorphism and incident HF. However, no cardiac imaging evaluation of the myocardial structure or function was performed [13]. Therefore, it remains unclear whether elevated levels of BNP, connected with rs198389 variants, may in fact contribute to more favorable cardiovascular outcomes in subjects at risk of HF. Concluding the EPIC-Norfolk study, Pfister et al. suggested a potential association between the *NPPB*:rs198389 polymorphism and individual subtypes of HF [14].

In the present study, possible associations among the *NPPB*:rs198389 polymorphism, NT-proBNP serum levels, and HF phenotypes have been investigated in a sample from the Polish population. We aimed to determine the possible contribution of the *NPPB*:rs198389 polymorphism to distinct HF phenotypes in Polish patients, as this polymorphism has not been studied in this population before. Past studies have examined different populations, yielding differing outcomes. We hypothesized that the *NPPB*:rs198389 polymorphism may be associated with increased NT-proBNP levels, as implied by some previous studies, which may contribute to the occurrence of distinct phenotypic features in patients diagnosed with HF.

## 2. Patients and Methods

### 2.1. Patients

This was a retrospective study of 250 consecutive patients aged 40–90 and diagnosed with chronic HF, who were referred for elective admission to the Departments of Cardiology or Cardiac Surgery at the Pomeranian Medical University in Szczecin, Poland, to determine possible treatment. Patients with acute concomitant illnesses that could substantially affect NT-proBNP levels were excluded. A total of 250 patients with chronic HF, New York Heart Association (NYHA) functional class I–IV (191 men and 59 women), were included in the study. HF in this group was caused by CAD, heart valve disease, or both, and they had been diagnosed from 6 months to 37 years before the study began. The group consisted of 118 patients with HFpEF (81 men and 37 women), 50 patients with HFmrEF (39 men and 11 women), and 82 patients with HFrEF (71 men and 11 women). Demographic data and medical history were collected from patients’ medical records. At the time of sampling, they were treated with medication according to the latest guidelines for HF therapy. All study participants underwent transthoracic echocardiography, and all patients routinely underwent NT-proBNP measurements to better assess the severity of HF. The serum concentration of NT-proBNP was measured using an electrochemiluminescence immunoassay (ECLIA) using commercially available kits and a Cobas E801 analyzer (both from Roche Diagnostics, Mannheim, Germany).

All patients were of Polish nationality, European descent, and lived in the Polish Western Pomerania region.

The study protocol approved by the Pomeranian Medical University Ethics Committee was performed according to the Declaration of Helsinki, and all participants provided formal informed consent.

### 2.2. Genotyping

Genomic DNA was isolated from whole blood using the QIAamp Blood DNA Mini Kit (QIAGEN, Hilden, Germany). Genotyping of the *NPPB*:rs198389 (c.-381T > C) polymorphism was carried out using polymerase chain reaction (PCR)-restriction fragment length polymorphism (PCR-RFLP) according to previously described methods [10]. All DNA samples were genotyped in a blind manner, i.e., the samples were anonymously labeled by one person and then genotyped by a second person. Briefly, amplification of a 500 bp *NPPB* sequence, including the rs198389 polymorphism, was performed by PCR using PCR Master Mix (2X) (Thermo Fisher Scientific, Waltham, MA, USA) with 5′-GCCGGGGCTGTTTTCGCTGTGAGT-3′ as the forward primer and 5′-CGGAGGCTGCTGCTGCTGCTTCTG-3′ as the reverse primer (from TIB MOL BIOL, Poznań, Poland). *NPPB* amplicons were subsequently digested with the EcoRII restriction enzyme (Thermo Fisher Scientific), yielding restriction fragments of 238, 133, 98, and 31 base pairs (bp) for the c.-381T allele or 371, 98, and 31 bp for the c.-381C allele. Restriction products were electrophoretically separated on 3% agarose gels and visualized by staining with Midori Green (Nippon Genetics Europe, Düren, Germany) (Figure S1). To verify the results, 10% of the DNA samples were randomly chosen and sequenced. (Figure S2). In each case, the results of *NPPB* genotyping obtained by sequencing were as expected based on the PCR-RFLP analysis.

### 2.3. Statistical Analyses

Possible deviation of *NPPB*:rs198389 genotype frequencies from the Hardy–Weinberg equilibrium was assessed using the chi-squared test. The size of the group to achieve a statistical power > 0.8 and significance of 0.05, assuming a mean of 2.5 and a standard deviation (SD) of 0.7 for log NT-proBNP (calculated from data), with a MAF ranging from 0.1 to 0.45 and differences among genotype classes from 0.14 to 0.56, was calculated according to Gauderman et al. [15]. Parameters were compared among groups using chi-squared or Fisher’s exact tests for qualitative variables and Kruskal–Wallis or Mann–Whitney tests for quantitative variables, as appropriate. The strength of associations of qualitative variables with genotypes and alleles was described as odds ratios with 95% confidence intervals (95%CI), whereas the strength of associations of quantitative variables with NT-proBNP terciles was described as an effect size (ES) with 95%CI. Two-tailed tests with *p* < 0.05 were considered statistically significant. If needed, *p*-values were adjusted for multiple comparisons using the Benjamini and Hochberg procedure to control for the false discovery rate (FDR). Studied associations were adjusted for potential confounding covariates. The significance of genetic components of the models was tested using a likelihood ratio test by comparing a full and a reduced model. All analyses were performed using RStudio (version 2023.03.1 + 446, Posit, Boston, MA, USA).

## 3. Results

The characteristics of patients are shown in Table 1. Body height, body mass, left ventricular mass index (LVMI), the frequency of left ventricular hypertrophy (LVH) or reduced EF, and creatinine concentration proved to be significantly higher amongst male patients when compared to females. Age, EF, and frequency of preserved EF were higher in women as compared to men (Table 1).

Among 250 HF patients, there were 92 TT homozygotes (36.8%), 119 TC heterozygotes (47.6%), and 39 CC homozygotes (15.6%), and the frequency of the minor *NPPB*:c.-381C allele was 39.4%. Among 59 HF females, there were 18 TT homozygotes (30.5%), 28 TC heterozygotes (47.5%), and 13 CC homozygotes (22.0%), and the frequency of the minor *NPPB*:c.-381C allele was 45.8%. Among 191 HF males, there were 74 TT homozygotes (38.8%), 91 TC heterozygotes (47.6%), 26 CC homozygotes (13.6%), and the frequency of the minor *NPPB*:c.-381C allele was 37.4%. There were 35 NYHA class I, 147 NYHA class II, 57 NYHA class III, and 11 NYHA class IV patients. There were no significant differences in the distribution of *NPPB* genotypes or alleles between HF females and HF males (*p* = 0.237 or *p* = 0.106, respectively). The *NPPB*:rs198389 genotype distributions in the HF group as well as in separately analyzed HF females or HF males were in Hardy–Weinberg equilibrium (*p* = 0.960, *p* = 0.735, or *p* = 0.813, respectively).

**Figure 1:**
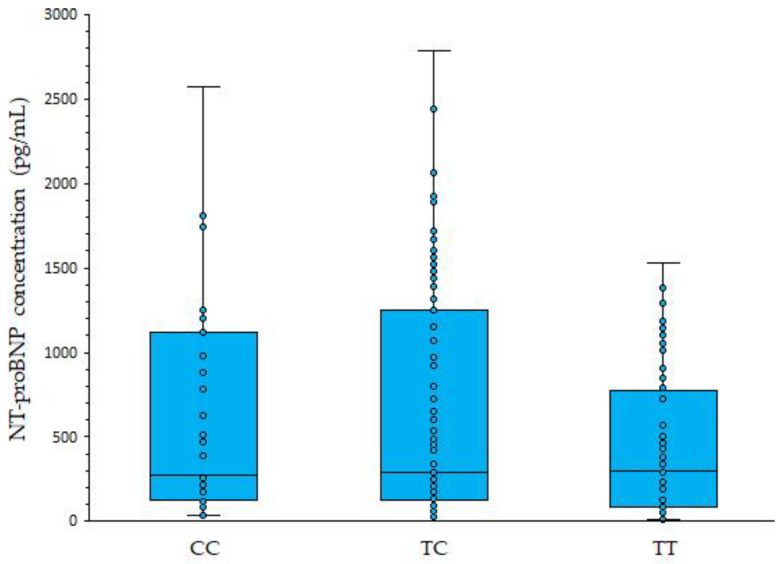
NT-proBNP concentrations in heart failure patients in regard to *NPPB*:rs198389 (c.-381T > C) genotypes. Box plots show medians, interquartile range and range (whiskers).

Except for body height, there were no significant differences in phenotypic features of HF patients in regard to *NPPB*:rs198389 genotypes (Table 2). Patients homozygous for the major *NPPB*:c.-381T allele were significantly taller as compared to subjects with at least one c.-381C allele (TT homozygotes were 3.4 cm (95%CI: 1.2:5.5 cm) taller than TC heterozygotes or CC homozygotes). However, this result might be incidental. There was no significant association between *NPPB*:rs198389 genotypes and NT-proBNP concentrations (Figure 1).

There were no significant differences in the distribution of either *NPPB*:rs198389 genotypes or alleles across terciles of NT-proBNP concentrations (Table 3).

Nevertheless, modest effects might be undetected in the presented group of patients. However, age, LVMI, C-reactive protein (CRP) levels, serum creatinine concentrations, and frequencies of MI, LVH, or reduced EF in patients from the lower tercile (LT) were significantly lower than in subjects from the middle (MT) and/or upper terciles (UT). In addition, body mass index (BMI) in the UT patients was significantly lower than in subjects from the LT of NT-proBNP concentrations. EF and the frequency of preserved EF in LT subjects were significantly higher compared to other groups (MT or UT patients) (Table 4 and Table 5).

## 4. Discussion

NT pro-BNP production is induced in response to ventricular myocardial stretch and presumably is modulated by promoter activity of its gene. The *NPPB*:rs198389 polymorphism influences transcriptional activity and is associated with varying NT-proBNP concentrations [11,16]. The findings of the present study confirm the previously established association between increased NT-proBNP production by cardiomyocytes in the heart ventricles and structural cardiac remodeling [17,18]. Although the association between NT-proBNP and cardiovascular diseases, especially HF, is clear and has been demonstrated in many studies, the association between *NPPB* polymorphisms and HF phenotype remains controversial. It therefore remains challenging to determine whether additional phenotypic factors attenuate the influence of *NPPB*:rs198389 variants on promoter responsiveness to transcription factors. Indeed, NT pro-BNP concentration might be more strongly influenced by these clinical features than by its own promoter genotype. Therefore, multivariable analyses incorporating potential confounders were included in the present study.

The present study has not demonstrated any crucial relationship between *NPPB*:rs198389 genotypes and phenotypic features of HF patients. In addition, within the HF group, there were no significant associations between the *NPPB*:rs198389 genotypes or alleles and the other analyzed variables except for height. There were no significant differences in the distribution of *NPPB*:rs198389 genotypes or alleles across NT-proBNP concentrations. Similar results were reported by Ichiki et al. [19]. In a study of 1146 patients from Minnesota, USA, the *NPPB*:rs198389 minor allele was not associated with any BP, metabolic, renal, or echocardiographic parameters in either sex [19]. Moreover, Johansson et al. did not associate *NPPB*:rs198389 genotypes with any tested phenotypic traits, including lipid profile, BP, or glycemia [20]. However, Meirhaeghe et al. found that the *NPPB*:rs198389 C allele was associated with higher BNP promoter activity and, consequently, higher BNP plasma concentrations [21]. Similar results were reported by Cannone et al. [12], although Cannone et al. investigated subjects at risk for HF from the STOP-HF study. They genotyped patients for the rs198389 polymorphism, assessed BNP levels, and determined clinical phenotypes. According to this study, HF patients carrying the rs198389 C allele had higher BNP levels [12]. Furthermore, they conducted a 5-year follow-up in which subjects without the considered rs198389 C allele had lower BNP concentrations, which was associated with higher cardiovascular risk. This suggests that higher circulating BNP levels may be associated with better cardiovascular outcomes in the long term. Compared to our study, they focused their results especially on the population at risk for HF, as well as on subjects with documented higher levels of BNP for an extended period of time [12].

The present study reported that higher NT-proBNP was associated with increased age, LVMI, CRP levels, serum creatinine concentrations, and the frequency of MI, LVH, or reduced EF. In addition, patients with higher NT-proBNP concentrations had lower BMI. Moreover, we showed that EF and the frequency of preserved EF were significantly higher amongst patients with lower NT-proBNP concentrations.

Our previous study has shown that the *NPPB*:rs198389 polymorphism is not associated with the risk of HF in Polish individuals [10]. Similar conclusions were reached by Pfister et al. during the EPIC-Norfolk study, in which researchers conducted a 12.6-year-long follow-up of 23 192 people. They did not find any significant association between rs198389 and HF risk [14]. Nevertheless, in both Pfister et al. and our study, a slight effect on HF course might be undetectable, so some small changes in HF phenotypes and overall HF risk cannot be entirely excluded. Available data, including our studies, on a potential association between *NPPB*:rs198389 variants and echocardiographic parameters, are incoherent [10,16,22].

It has been observed that the *NPPB*:rs198389 CC homozygotes were associated with higher BP [16]. Moreover, an association between *NPPB*:rs198389 and systolic BP was reported for African American subjects [22]. Nevertheless, in a group of patients from New Zealand, *NPPB*:rs198389 was not associated with the occurrence of HTN [23]. What is more, according to Cannone et al., HF patients bearing the rs198389 C allele were at lower risk of HTN [12].

In a 23-year-long follow-up of 11 361 patients from various ethnic groups, the CC genotype was associated with a higher mean plasma NT-proBNP level than the TT genotype, with heterozygotes showing intermediate values. The CC genotype was also associated with lower BP and reduced antihypertension medication use compared with the TT genotype, again with intermediate values in heterozygotes. CC genotype subjects had lower mortality, primarily reflective of cardiovascular death [11]. The Seidelmann et al. study consisted of biracial participants aged 45–64 years from four US communities, in comparison to our smaller group of single-race Polish participants in a larger age range between 40 and 90 years. The disparities in the studied cohorts could lead to different results in these two studies [11]. Another study, conducted among Kurdish people in Iraq, suggested a potential association between the CC genotype and a reduced risk of CAD [24]. According to Cannone et al., left ventricular systolic dysfunction and hospitalization for MACE happened less often in patients with the *NPPB*:rs198389 C allele [12]. Similar results were reported by Li et al., but only for women [25]. In a group of 513 patients undergoing clinically indicated coronary angiography, the frequency of the *NPPB*:rs198389 TT genotype was significantly higher in women with angiographic evidence for obstructive CAD [25]. According to Wu et al., *NPPB*:rs198389 is associated with reduced risk of left ventricular dysfunction in CAD patients with hyperlipidemia [26]. Nevertheless, Johansson et al. did not demonstrate any significant association between *NPPB*:rs198389 and clinical endpoints in a CAD trial. None of the *NPPB*:rs198389 genotypes or alleles were associated with MI, stroke, or cardiovascular death [20].

In 2007, Meirhaeghe et al. showed that the *NPPB*:rs198389 CC genotype is associated with lower fasting glucose levels compared to other genotypes. Moreover, individuals with this genotype were less likely to develop type 2 DM (T2DM) [21]. Results were further replicated in four case–control studies [21]. A meta-analysis of 49 279 Europeans confirmed a previously suspected association between the *NPPB*:rs198389 CC genotype and a lower risk of T2DM [27]. This was again confirmed in another meta-analysis by Pfister et al. [28]. A similar result was reported in a prospective case–cohort study [29]. However, in a population of different ethnicities, i.e., in an Algerian population, results were different [30].

In the Polish population of patients with atherosclerotic renovascular HTN, *NPPB*:rs198389 C allele homozygotes were more likely to develop atherosclerotic lesions in renal arteries and high-grade renal artery stenosis [31].

The major limitations of our study are the relatively small size of the group and the complex pathology of HF, depending on widespread traditional environmental risk factors such as smoking and obesity. It has previously been demonstrated that the direct influence of the local environment modulates genetic risk, reducing the ability to draw reliable conclusions [32]. Nevertheless, during the EPIC Norfolk study, researchers did not prove an association between *NPPB*:rs198389 and HF even after stratification by HTN, obesity, and CAD [14].

## 5. Conclusions

The results of our study did not confirm a relationship between the *NPPB*:rs198389 gene polymorphism and HF phenotypes in the Polish population. Further reproduction studies in the ethnic groups, in which a putative relationship has been shown (precisely following the relevant research-group methods), are warranted, especially in order to eliminate potential false positives.

Due to the complexity of HF and the influence of multiple environmental risk factors, a larger cohort study is needed to fully understand this possible association. According to the aforementioned studies, *NPPB* genetic variants might influence NT-proBNP concentration, which corresponds with HF phenotypes. Further research would lead to a better comprehension of the underlying mechanisms of HF etiology. It might be crucial to identify novel genetic factors contributing to HF development and potential BNP-based therapies to prevent HF.

## Figures and Tables

**Table 1:** Characteristics of the heart failure patients with regard to sex.

Variable	All (n = 250)	Females (n = 59)	Males (n = 191)	*p* ^a^
Age (years)	66 (61–70)	69 (64–74)	65 (60–69)	0.001
Body height (m)	1.69 (1.63–1.73)	1.58 (1.52–1.61)	1.70 (1.67–1.76)	0.001
Body mass (kg)	81 (72–91)	71 (64–79)	83 (75–94)	0.001
BMI (kg/m^2^)	28.9 (26.1–31.6)	29.0 (26.2–32.0)	28.7 (26.1–31.5)	0.567
BMI ≥ 30 kg/m^2^, n (%)	96 (38)	27 (46)	69 (36)	0.221
Smokers, n (%)	93 (37)	23 (39)	70 (37)	0.760
Myocardial infarction, n (%)	130 (52)	31 (53)	99 (52)	0.999
Arterial hypertension, n (%)	212 (85)	54 (92)	158 (83)	0.145
Diabetes, n (%)	93 (37)	21 (36)	72 (38)	0.878
LVH, n (%)	130 (52)	39 (66)	91 (48)	0.017
LVMI, g/m^2^	111 (93–137)	104 (84–127)	112 (94–139)	0.043
EF, %	45 (40–55)	50 (45–55)	45 (40–55)	0.003
pEF, n (%)	118 (47)	37 (63)	81 (42)	0.007
mrEF, n (%)	50 (20)	11 (19)	39 (20)	0.766
rEF, n (%)	82 (33)	11 (19)	71 (37)	0.011
NYHA III–IV, n (%)	68 (27)	15 (25)	53 (28)	0.867
CRP (mg/L)	2 (1–5)	3 (1–5)	2 (1–5)	0.613
Creatinine (mg/dL)	0.87 (0.78–1.00)	0.80 (0.71–0.94)	0.88 (0.80–1.02)	0.001
NT-proBNP [pg/mL]	289 (109–996)	308 (125–850)	287 (89–1069)	0.695
NT-proBNP (log)	2.46 (2.04–3.00)	2.49 (2.10–2.93)	2.46 (1.95–3.03)	0.695

Quantitative variables are presented as median, lower quartile and upper quartile (Q1:Q3). Qualitative data are presented as a number with corresponding percentage. ^a^ Mann–Whitney tests for quantitative variables and Fisher’s exact tests for qualitative variables; BMI—body mass index; CRP—C-reactive protein; EF—ejection fraction; mrEF—mildly reduced ejection fraction; pEF—preserved ejection fraction; rEF—reduced ejection fraction; LVH—left ventricular hypertrophy; LVMI—left ventricular mass index; NT-proBNP—N-terminal pro-B-type natriuretic peptide; NYHA—New York Heart Association functional classes I–IV; log—logarithm.

**Table 2:** Characteristics of the heart failure patients in regard to the *NPPB*:rs198389 (c.-381T > C) polymorphism.

Variable	*NPPB*:rs198389 (c.-381T > C) Genotype			*p* ^a^	*p _D_* ^b^	*p _R_* ^b^	*p _TT vs. CC_* ^b^
	TT (n = 92)	TC (n = 119)	CC (n = 39)				
Male, n (%)	74 (80)	91 (76)	26 (67)	0.241	0.282	0.150	0.116
Age (years)	66 (61–69)	65 (62–71)	67 (60–71)	0.741	0.472	0.976	0.776
Body height (m)	1.70 (1.64–1.75)	1.67 (1.61–1.73)	1.67 (1.61–1.74)	0.070	0.022	0.602	0.176
Body mass (kg)	82 (73–90)	81 (71–92)	76 (71–89)	0.605	0.646	0.322	0.325
BMI (kg/m^2^)	28.4 (25.6–30.9)	29.1 (26.3–32.1)	28.8 (24.5–31.3)	0.159	0.223	0.318	0.809
BMI ≥ 30 kg/m^2^, n (%)	30 (33)	53 (45)	13 (33)	0.168	0.178	0.591	0.999
Smokers, n (%)	33 (36)	45 (38)	15 (38)	0.952	0.787	0.859	0.844
Myocardial infarction, n (%)	48 (52)	63 (53)	19 (49)	0.926	0.999	0.728	0.849
Arterial hypertension, n (%)	79 (86)	98 (82)	35 (90)	0.572	0.855	0.469	0.777
Diabetes, n (%)	35 (38)	45 (38)	13 (33)	0.892	0.892	0.719	0.694
LVH, n (%)	42 (46)	68 (57)	20 (51)	0.252	0.149	0.999	0.572
LVMI (g/m^2^)	105 (93–136)	117 (93–138)	107 (94–137)	0.687	0.442	0.902	0.807
EF (%)	45 (40–55)	45 (40–55)	45 (40–55)	0.709	0.518	0.783	0.951
pEF, n (%)	45 (49)	55 (46)	18 (46)	0.926	0.695	0.999	0.849
mrEF, n (%)	20 (22)	23 (19)	7 (18)	0.856	0.600	0.727	0.624
rEF, n (%)	27 (29)	41 (34)	14 (36)	0.649	0.404	0.711	0.537
NYHA III-IV, n (%)	27 (29)	34 (29)	7 (18)	0.366	0.559	0.176	0.198
CRP (mg/L)	2 (1–5)	2 (1–6)	2 (1–5)	0.482	0.233	0.826	0.496
Creatinine (mg/dL)	0.88 (0.79–0.97)	0.87 (0.77–1.06)	0.85 (0.77–0.93)	0.412	0.640	0.184	0.177
NT-proBNP (pg/mL)	296 (85–760)	289 (125–1252)	273 (122–1121)	0.359	0.154	0.709	0.357
NT-proBNP (log)	2.47 (1.93–2.88)	2.46 (2.10–3.10)	2.44 (2.09–3.05)	0.359	0.154	0.710	0.357
NT-proBNP (log) after adjustment for age, sex, creatinine concentration, LVMI, EF, and BMI				0.353	0.222	0.257	0.721

Quantitative variables are presented as median, lower quartile and upper quartile (Q1:Q3). Qualitative data are presented as a number with corresponding percentage. ^a^ Kruskal–Wallis test for quantitative variables and chi-square test for qualitative variables; ^b^ Mann–Whitney test for quantitative variables and Fisher’s exact test for qualitative variables; BMI—body mass index; CRP—C-reactive protein; EF—ejection fraction; mrEF—mildly reduced ejection fraction; pEF—preserved ejection fraction; rEF—reduced ejection fraction; LVH—left ventricular *p* _D_—*p* value in the dominant inheritance model of c.-381C allele; *p* _R_—*p* value in the recessive inheritance model of c.-381C allele; *p* _TT vs. CC_—*p* value for comparison of TT homozygotes with CC ones. For other abbreviations, see Table 1.

**Table 3:** Frequency distribution of *NPPB*:rs198389 (c.-381T > C) genotypes or alleles in heart failure patients in regard to terciles of NT-proBNP concentrations.

*NPPB*:rs198389 Polymorphism	NT-proBNP Concentration [pg/mL]			*p _LT vs. MT vs. UT_ * ^a^	*p _LT + MT_* *_vs. UT_* ^b^	*p _LT vs._* *_MT + UT_* ^b^	*p _LT vs. UT_ * ^b^
n (%)	LT: ≤175 (n = 84)	MT: 177–619 (n = 82)	UT: ≥628 (n = 84)				
TT	33 (39)	35 (43)	24 (29)	0.414	0.158	0.823	0.336
TC	39 (47)	35 (43)	45 (53)				
CC	12 (14)	12 (14)	15 (18)				
TT	33 (39)	35 (43)	24 (29)	0.143	0.055	0.562	0.142
TC + CC	51 (61)	47 (57)	60 (71)				
TT + TC	72 (86)	70 (86)	69 (82)	0.781	0.484	0.684	0.529
CC	12 (14)	12 (14)	15 (18)				
T	105 (62)	105 (64)	93 (55)	0.224	0.088	0.536	0.183
C	63 (38)	59 (36)	75 (45)				

^a^ Chi-square test; ^b^ Fisher’s exact test; *p* _LT vs. MT vs. UT_—*p* value for comparison of LT subjects, MT subjects, and UT subjects; *p* _LT + MT vs. UT_—*p* value for comparison of LT + MT with UT subjects; *p* _LT vs. MT + UT_—*p* value for comparison of LT with MT + UT subjects; *p* _LT vs. UT_—*p* value for comparison of LT with UT subjects; UT—upper tercile. For other abbreviations, see Table 1.

**Table 4:** Characteristics of heart failure patients in regard to terciles of NT-proBNP concentration.

Variable	NT-proBNP Concentration [pg/mL]			*p _LT vs. MT vs. UT_* ^a^	*p _LT + MT vs. UT_* ^b^	*p _LT vs. MT + UT_* ^b^	*p _LT vs. UT_* ^b^
	LT: ≤175 (n = 84)	MT: 177–619 (n = 82)	UT: ≥628 (n = 84)				
Male, n (%)	65 (77)	61 (74)	65 (77)	0.872	0.875	0.875	1.000
Age (years)	65 (60–68)	67 (62–71)	66 (61–71)	0.095	0.517	0.035	0.119
Body height (cm)	169 (164–174)	168 (163–173)	168 (162–174)	0.922	0.788	0.694	0.718
Body mass (kg)	83 (73–94)	80 (71–90)	80 (71–90)	0.221	0.236	0.090	0.099
BMI (kg/m^2^)	29.2 (26.9–32.1)	29.0 (25.8–31.7)	28.1 (25.5–31.2)	0.124	0.082	0.071	0.037
BMI ≥ 30 kg/m^2^, n (%)	36 (43)	30 (37)	30 (36)	0.584	0.583	0.336	0.430
Smokers, n (%)	30 (36)	28 (34)	35 (42)	0.570	0.333	0.783	0.526
Myocardial infarction, n (%)	28 (33)	44 (54)	58 (69)	2.0 × 10^−5^	0.001	3.0 × 10^−5^	6.2 × 10^−6^
Arterial hypertension, n (%)	72 (86)	71 (87)	69 (82)	0.699	0.457	0.853	0.675
Diabetes, n (%)	29 (35)	27 (33)	37 (44)	0.275	0.128	0.581	0.269
LVH, n (%)	32 (38)	37 (45)	61 (73)	1.4 × 10^−5^	4.2 × 10^−6^	0.003	1.1 × 10^−5^
LVMI (g/m^2^)	98 (85 –116)	109 (93–130)	131 (106–156)	1.3 × 10^−7^	3.9 × 10^−7^	2.9 × 10^−6^	5.1 × 10^−8^
EF (%)	55 (50–55)	45 (40–50)	40 (29–45)	9.7 × 10^−16^	5.9 × 10^−13^	4.9 × 10^−13^	1.4 × 10^−14^
pEF, n (%)	64 (76)	37 (45)	17 (20)	3.1 × 10^−12^	8.2 × 10^−10^	5.3 × 10^−11^	2.9 × 10^−13^
mrEF, n (%)	15 (18)	21 (26)	14 (17)	0.296	0.405	0.617	1.000
rEF, n (%)	5 (6)	24 (29)	53 (631)	2.2 × 10^−14^	1.1 × 10^−12^	5.4 × 10^−12^	8.9 × 10^−16^
CRP (mg/L)	1 (1–3)	2 (1–5)	3 (1–11)	0.004	0.002	0.007	0.002
Creatinine (mg/dL)	0.83 (0.76–0.94)	0.87 (0.77–0.96)	0.94 (0.8–1.23)	9.3 × 10^−5^	2.3 × 10^−5^	0.005	6.5 × 10^−5^

Quantitative variables are presented as median, lower quartile and upper quartile (Q1:Q3). Qualitative data are presented as a number with corresponding percentage. ^a^ Kruskal–Wallis test for quantitative variables and chi-square test for qualitative variables; ^b^ Mann–Whitney test for quantitative variables and Fisher’s exact test for qualitative variables; BMI—body mass index; CRP—C-reactive prop _LT vs. MT vs UT_—*p* value for comparison of LT subjects, MT subjects, and UT subjects; *p* _LT + MT vs. UT_—*p* value for comparison of LT + MT with UT subjects; *p* _LT vs. MT + UT_—*p* value for comparison of LT with MT + UT subjects; *p* _LT vs. UT_—*p* value for comparison of LT with UT subjects; UT—upper tercile.

**Table 5:** Effect sizes for associations between terciles of NT-proBNP concentration and heart failure phenotypes.

Variable	*LT + MT vs. UT*		*LT vs. MT + UT*		*LT vs. UT*	
	*p* ^a^	ES (95% CI)	*p* ^a^	ES (95% CI)	*p* ^a^	ES (95% CI)
Age (years)	0.517	−0.10 (−0.32, 0.12)	0.035	−0.30 (−0.52, −0.08)	0.119	−0.27 (−0.52, −0.01)
BMI (kg/m^2^)	0.082	0.24 (0.02, 0.46)	0.071	0.24 (0.02, 0.46)	0.037	0.33 (0.07, 0.58)
LVMI (g/m^2^)	3.9 × 10^−7^	−0.78 (−1.0, −0.55)	2.9 × 10^−6^	−0.63 (−0.85, −0.40)	5.1 × 10^−8^	−0.88 (−1.1, −0.61)
EF (%)	5.9 × 10^−13^	1.2 (0.92, 1.4)	4.9 × 10^−13^	1.0 (0.78, 1.2)	1.4 × 10^−14^	1.4 (1.2, 1.7)
CRP (mg/L)	0.002	−0.35 (−0.57, −0.13)	0.007	−0.26 (−0.48, −0.04)	0.002	−0.37 (−0.62, −0.11)
Cr (mg/dL)	2.3 × 10^−5^	−0.49 (−0.71, −0.26)	0.005	−0.27 (−0.49, −0.05)	6.5 × 10^−5^	−0.42 (−0.68, −0.16)

ES = effect size. ^a^ Mann–Whitney test for quantitative variables and Fisher’s exact test for qualitative variables. For other abbreviations, see Table 1.

## Data Availability

The data presented in this study are available on request from the corresponding author.

## Supplementary Materials

The following supporting information can be downloaded at: https://www.mdpi.com/article/10.3390/genes17040454/s1, Figure S1: Gel electrophoresis of *NPPB*:rs198389 amplicons after digestion with *Eco*RII; Figure S2: Sequencing chromatograms for *NPPB*:rs198389 TT homozygote (upper panel), CT heterozygote (middle panel) and CC homozygote (lower panel).
