# Supplementary material for: Association Analyses Between the NPPB:rs198389 Gene Polymorphism, NT-proBNP Serum Concentrations and Phenotypic Features in Patients with Heart Failure
Source: Genes (Basel). 2026 Apr 14;17(4):454. doi: 10.3390/genes17040454 (PMC13116900; doi:10.3390/genes17040454)
Supplement: Supplementary file 1 [file genes-17-00454-s001.zip › genes-4100351-Figure S1.pdf]

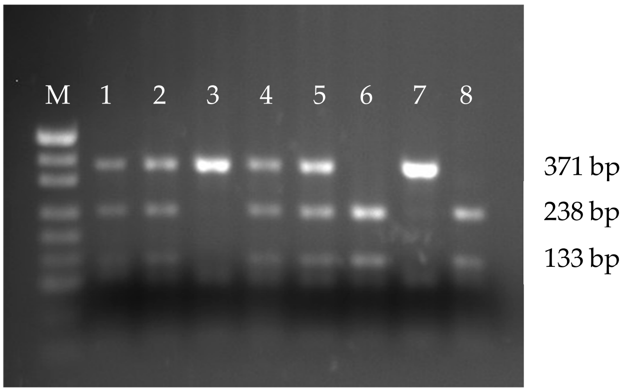

Figure S1. Gel electrophoresis of *NPPB*:rs198389 amplicons after digestion with *Eco*RII

Lanes:
M – pUC 19DNA molecular size marker (Thermo Fisher Scientific)
1, 2, 4, 5 – CT heterozygote
3, 7 – CC homozygote
6, 8 – TT homozygote
