# Supplementary material for: Association Analyses Between the NPPB:rs198389 Gene Polymorphism, NT-proBNP Serum Concentrations and Phenotypic Features in Patients with Heart Failure
Source: Genes (Basel). 2026 Apr 14;17(4):454. doi: 10.3390/genes17040454 (PMC13116900; doi:10.3390/genes17040454)
Supplement: Supplementary file 1 [file genes-17-00454-s001.zip › genes-4100351-Figure S2.pdf]

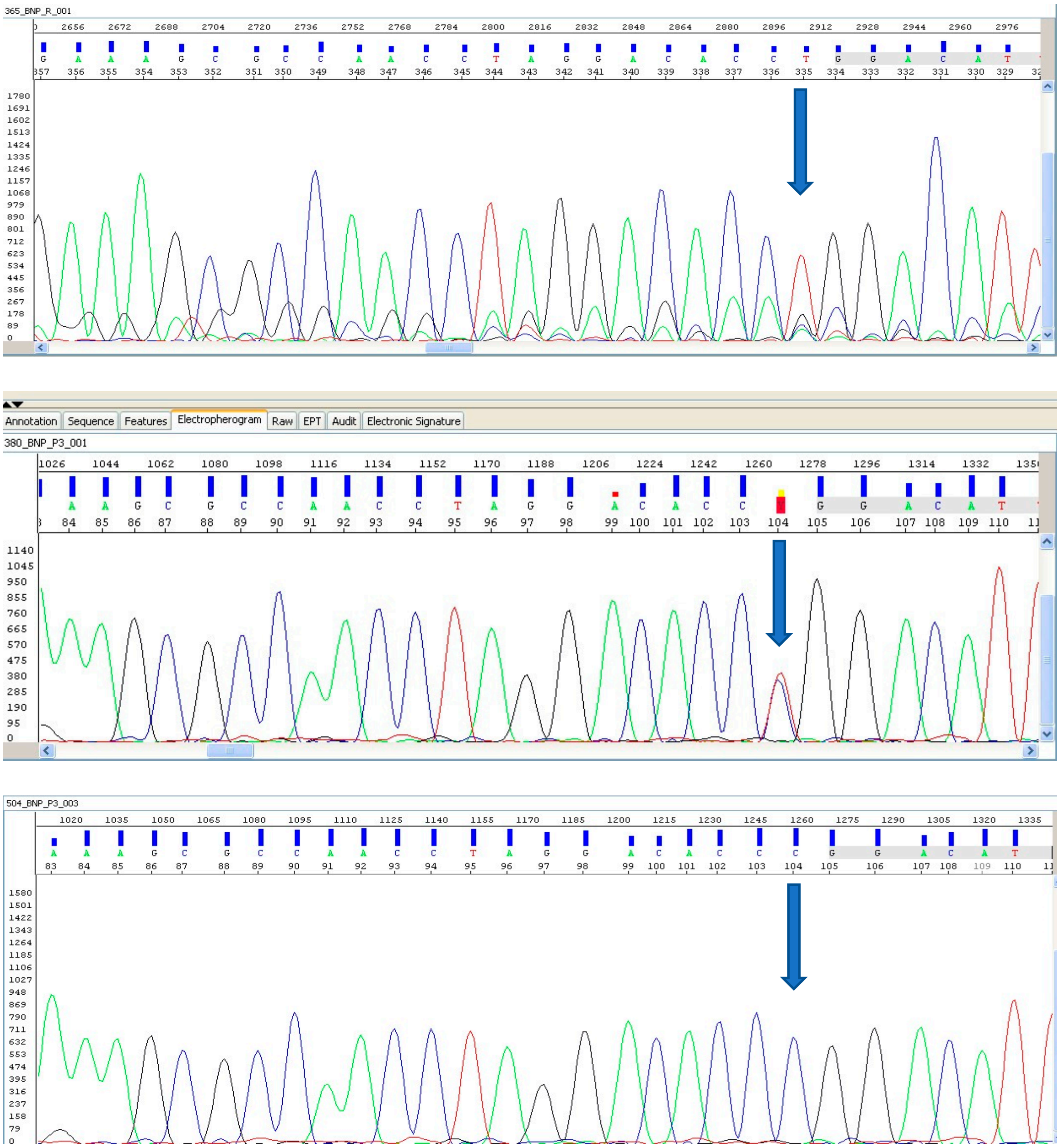

Figure S2. Sequencing chromatograms for *NPPB*:rs198389 TT homozygote (upper panel), CT heterozygote (middle panel) and CC homozygote (lower panel).
